# Effectiveness of Microscopic Tubular Discectomy for Improved Pain and Mobility in Far Lateral Lumbar Disc Herniation: A Systematic Review

**DOI:** 10.1111/os.70187

**Published:** 2025-10-12

**Authors:** Chamath Jagoda, Samantha Spanos, Timothy L. Siu

**Affiliations:** ^1^ Macquarie Medical School, Faculty of Medicine, Health and Human Sciences Macquarie University Sydney Australia; ^2^ Australian Institute of Health Innovation, Faculty of Medicine, Health, and Human Sciences Macquarie University Sydney Australia; ^3^ Department of Clinical Medicine, Faculty of Medicine, Health and Human Sciences Macquarie University Sydney Australia

**Keywords:** extra foraminal lumbar disc herniation, far lateral lumbar disc herniation, microdiscectomy, microscopic tubular discectomy, minimally invasive surgery, systematic review

## Abstract

Far lateral lumbar disc herniation (FLLDH) is a subtype of lumbar disc herniation marked by severe radicular and lower back pain, often accompanied by sensory and motor dysfunction. Microscopic tubular discectomy (MTD) is a contemporary minimally invasive approach for treating FLLDH, yet its superiority over conventional discectomy remains inconclusive. The purpose of this systematic review was to assess the effectiveness of MTD in improving pain and mobility for FLLDH patients. A secondary aim was to assess the safety of MTD. Primary outcome measures were patient‐reported pain, assessed using a visual analog scale (VAS), patient‐reported mobility, assessed using the Oswestry Disability Index (ODI), and scores on the Modified MacNab criteria clinical assessment. Secondary outcome measures were mean blood loss, operation duration, hospital stay, reherniation rate, reoperation rate, and any peri‐ or postoperative complications. MEDLINE, Embase, and Scopus were searched for empirical studies on MTD for FLLDH, reporting pain or mobility outcomes. Data extracted included study design, participant characteristics, pre‐ and postmean scores for pain and mobility, blood loss, operation time, hospitalization duration, reherniation rate, and complications. Articles were quality appraised using Joanna Briggs Institute (JBI) quality appraisal tools. Of 271 articles identified, 15 were included. Preoperative leg pain scores ranged from 5.5 to 8.6, and postoperative scores ranged from 1.2 to 3.8. For lower back pain, preoperative scores ranged from 2.4 to 7.6, with postoperative scores from 1 to 4.8. Seven studies assessed mobility using the ODI, reporting significant improvements; pre‐operative scores ranged from 30.6 to 56.7, and postoperative scores ranged from 5.5 to 30.3. Seven studies used the Modified MacNab criteria, reporting excellent outcomes in 18.2%–71% of patients, good in 23%–54.5%, fair in 0%–18.2%, and poor in 0%–18%. Mean blood loss ranged from 30 to 70 mL, mean operation times from 43 to 126 min, and average hospital stays were, on average, ≤ 4 days. Reherniation was minimal, and the reoperation rate was low. Complications were rare, primarily transient neuropraxic symptoms. This review comprehensively synthesized empirical research on the effectiveness and safety of MTD for treating FLLDH. Overall, the findings indicate that MTD can reduce pain and improve mobility in patients with FLLDH, but limited sample sizes, variable follow‐up periods, and a lack of controlled studies constrain definitive conclusions about MTD's superiority over other discectomy techniques.

**PROSPERO registration number:** CRD42023443900

## Graphical abstract

Microscopic tubular discectomy for far lateral lumbar disc herniation significantly reduces radicular and lower back pain, improves functional mobility, and involves minimal blood loss, short hospital stays, and rare transient complications.

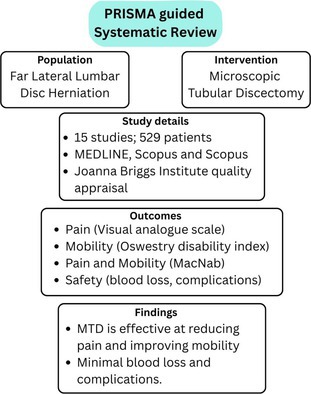

**Abbreviations**
ED: endoscopic discectomy
FLLDH: far lateral lumbar disc herniation
JBI: Joanna Briggs Institute
LDH: lumbar disc herniation
MTD: microscopic tubular discectomy
ODI: Oswestry Disability Index
PRISMA: Preferred Reporting Items for Systematic Reviews and Meta‐Analyses guidelines
VAS: visual analog scale

## 1 Background

Far lateral lumbar disc herniation (FLLDH) is a subtype of lumbar disc herniation (LDH), accounting for 6.5%–12% of all lumbar disc herniations [1]. FLLDH compresses the exiting nerve root and the corresponding dorsal root ganglion in the foraminal and extraforaminal zones. This compression can result in extreme leg pain, lower back pain, and sensory and motor dysfunction, which are often refractory to conservative therapies [2, 3, 4].

**FIGURE 1:**
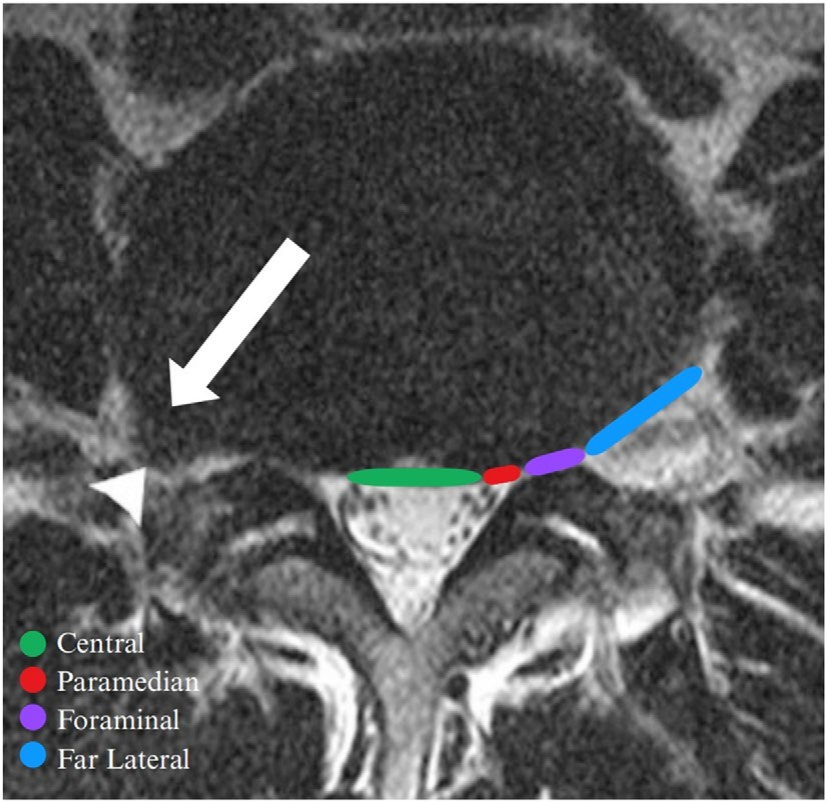
Schematic representation of the typical locations of lumbar disc herniations in an axial *T*_2_ weighted image. Green: central, red: paramedian, purple: foraminal, blue: far lateral. The arrow highlights a right‐sided far lateral disc herniation at the L4–L5 level, demonstrating asymmetrical disc contour.

**FIGURE 2:**
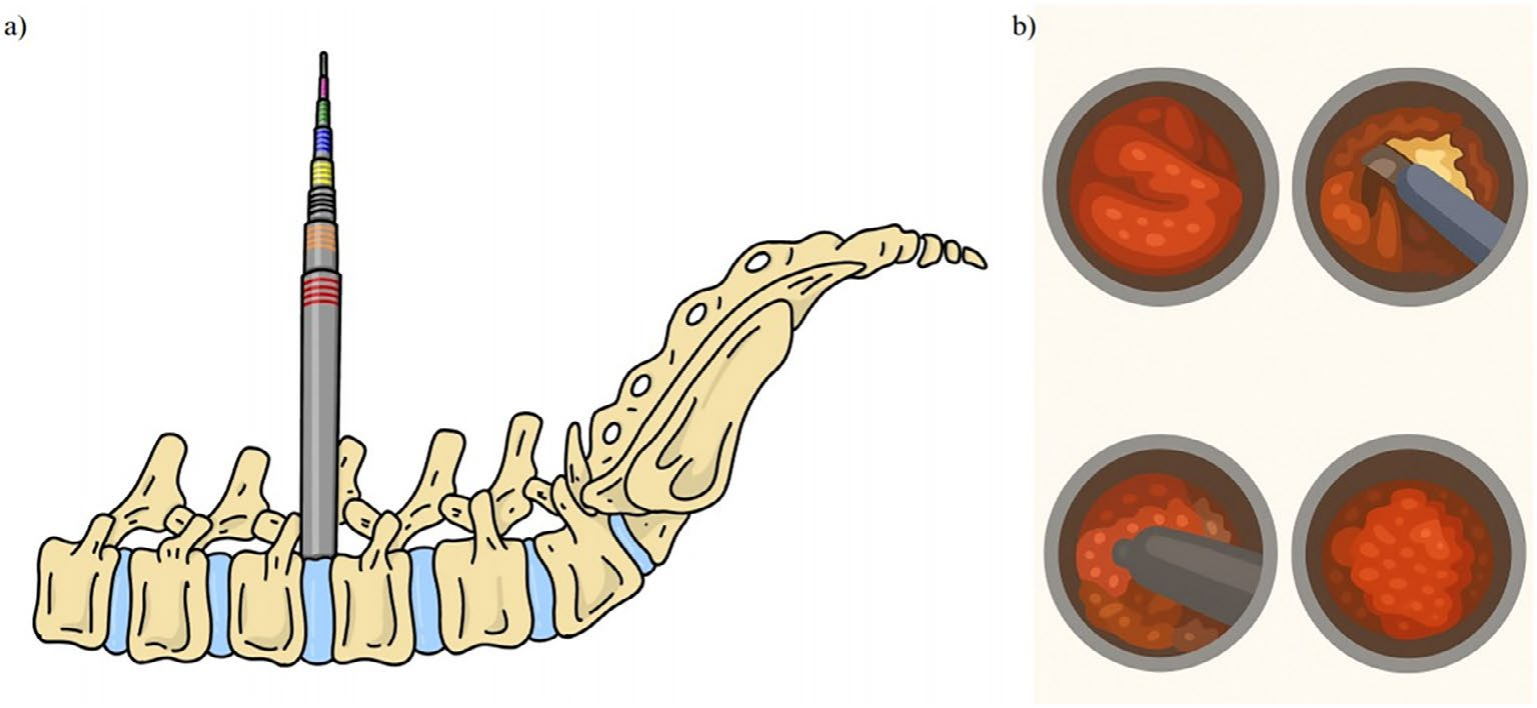
Schematic of the MTD technique for FLLDH. (a) Sequential placement of dilators to create a paraspinal surgical corridor, culminating in seating the tubular retractor on the spinal anatomy. (b) Microscopic view through the tubular retractor of the surgical corridor.

Microscopic tubular discectomy (MTD) is a minimally invasive paraspinal approach for the treatment of FLLDH. It has been previously demonstrated that MTD can reduce morbidity, accelerate recovery, and shorten hospital stays when compared to conventional discectomy [5, 6, 7, 8, 9, 10, 11, 12]. Despite these preliminary findings, no systematic review has been conducted to consolidate and critically assess the current body of evidence on the effectiveness of MTD for FLLDH. This systematic review aims to identify empirical studies on MTD for treating FLLDH and assess the range and distribution of observed effects on pain and mobility. A secondary aim was to assess the safety of MTD by synthesizing procedural metrics (e.g., operation time) and physical outcomes (e.g., blood loss) (Figures 1 and 2).

## 2 Methods

This systematic review was conducted and reported in accordance with the Preferred Reporting Items for Systematic Reviews and Meta‐Analyses guidelines (PRISMA) [13].

### 2.1 Search Strategy

Comprehensive search strategies were developed in collaboration with a medical librarian from Macquarie University using key words related to MTD and FLLDH (Appendix A in the Supporting Information). Literature searches were conducted in MEDLINE, Embase, and Scopus on July 24, 2023, and updated on the March 23, 2024. In addition to database searches, the reference lists of systematic reviews were snowballed to identify additional primary research studies that met eligibility criteria.

### 2.2 Study Selection

References were uploaded into Rayyan screening software where duplicates were identified and removed [14]. Two team members (C.J., S.S.) independently double screened the titles and abstracts of all references against the inclusion and exclusion criteria (Table 1). Discrepancies in decision‐making were resolved through discussion between the team members. Articles included during the title and abstract screening phase were independently read in full by C.J. and S.S. and assessed for eligibility against inclusion and exclusion criteria (Table 1). Any disparities between C.J. and S.S. were resolved through discussion, with T.S. available for resolution if needed.

**TABLE 1:** Inclusion and exclusion criteria for study selection.

Inclusion criteria	Exclusion criteria
Studies reporting on MTD technique; had to utilize tubular retractors and an operating microscope in the procedure. Studies reporting on patients with FLLDH (confirmed via medical imaging). Studies reporting preoperative scores and at least one postoperative score on the VAS, ODI, and/or Modified MacNab criteria. Full‐text empirical articles. Articles available in English. Adult patients (18+).	Studies not reporting on MTD. Studies not specifying the patient population as FLLDH or not providing information on the type of LDH. Studies not reporting pre‐operative and post‐operative scores on the VAS, ODI, or MacNab criteria. Nonempirical articles (editorials, reviews, opinions, and commentary articles). Case reports (providing data on one patient).

Abbreviations: FLLDH, far lateral lumbar disc herniation; LDH, lumbar disc herniation; MTD, microscopic tubular discectomy; ODI, Oswestry Disability Index; VAS, visual analog scale.

### 2.3 Data Extraction

Data were extracted from included articles in an excel spreadsheet created by C.J. and S.S., which was pilot tested with a small subset of articles and iteratively refined until fit for purpose. Extracted data were checked for agreement between authors. In instances where required data were not reported in studies, corresponding authors were contacted to obtain the missing data.

For study characteristics, extracted data included study authors, year and country of publication, study design, comparator or control group (if available), participant sample size, age, gender, and herniated disc level. For primary outcomes, the extracted data included preoperative, postoperative, and follow‐up scores on the visual analog scale (VAS) for leg and back pain, the Oswestry Disability Index (ODI), and the Modified MacNab criteria, along with the measurement timepoints. For secondary outcomes, extracted data included (where available) surgery duration (in minutes), reherniation rate, reoperation rate, duration of hospital stay (in days), blood loss (in mL), and details about surgical complications.

### 2.4 Quality Appraisal

The Joanna Briggs Institute (JBI) Critical Appraisal Checklists for Case Series and Cohort Studies were used to assess the quality of all studies [15]. The case series checklist evaluates article quality across 10 aspects of study design and reporting, while the cohort study checklist covers 11 aspects. Each criterion is assessed with responses of “yes,” “no,” “unclear,” or “not applicable.” Each article was independently assessed by C.J. and S.S., and consensus on quality ratings was reached via discussion between the study authors.

### 2.5 Synthesis Methods

Extracted data were narratively synthesized through textual descriptions, tabulation, and descriptive statistical analysis [16]. Quantitative outcomes were grouped based on the type of outcome measured (i.e., pain, mobility, Macnab criteria assessment). For primary outcomes, the range of mean preoperative, postoperative, and follow‐up scores was calculated across all studies for each outcome measure. For secondary outcomes, the range of mean operation time (min), blood loss (mL), hospital duration (days), reoperation, and reherniation (proportion of patient sample), and complications (proportion of patient sample) was calculated where possible. Where no means were provided for secondary outcomes, the range of raw scores within each study was tabulated.

## 3 Results

### 3.1 Study Selection

**FIGURE 3:**
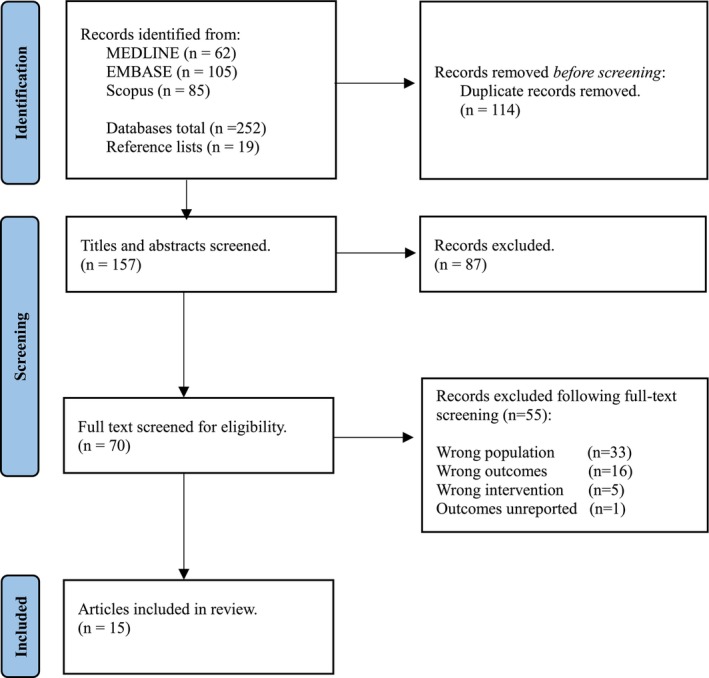
PRISMA flowchart outlining the screening process.

Figure 3 presents the PRISMA flowchart outlining the screening process. Database searches yielded 252 articles, and 19 articles were identified through snowballing. From those 252 records, 114 duplicates were detected and subsequently removed, leaving 157 records. Of the 157 records, 87 studies were excluded at title and abstract screening, leaving 70 records. During full‐text screening, 55 records were excluded for failing to meet eligibility criteria (Figure 3), and 15 articles were eligible for inclusion in the current review.

### 3.2 Study Characteristics

The characteristics of included articles are displayed in Table 2. The total number of participants across all included articles was 529 patients, with individual sample sizes ranging from 15 to 76 participants, and the gender distribution was 54% male and 46% female. The weighted average age across all studies, except in one study that did not report mean age, was 57.1 years; the standard deviation was reported in only five studies. Most studies were conducted in Germany (*n* = 6), followed by Australia (*n =* 3). Fourteen studies were case series, of which eight were retrospective and seven were prospective. One study was a retrospective cohort study.

**TABLE 2:** Characteristics of included studies and participants.

Author (year)	Country	Design	Sample size	Mean age in years (SD)	Male/female	Herniated disc level
Abdelgawaad. (2018) [17]	Germany	Prospective case‐series	76	54 (12)	41/35	L3/L4 L4/L5 L5/S1
Antony (2022) [18]	Australia	Prospective case‐series	28	55.93 (14.5)	15/13	L5/S1
Eicker (2013) [19]	Germany	Prospective case‐series	51	56.6	29/22	L3/L4 L4/L5 L5/S1
Fuentes (2009) [20]	France	Retrospective case‐series	26	56	15/11	L1/L2 L2/L3 L3/L4 L4/L5 L5/S1
Greiner‐Perth (2003) [5]	Germany	Retrospective case‐series	15	60.3	7/8	L5/S1
Hitchon (2015) [21]	USA	Retrospective case‐series	40	58 (12)	21/19	L1/L2 L2/L3 L3/L4 L4/L5 L5/S1
Kang (2024) [22]	Korea	Retrospective cohort study	33	56.82 (16.30)	20/13	L3/L4 L4/L5 L5/S1
Kogias (2007) [23]	Germany	Retrospective case‐series	38	61.5	^a^	L2/L3 L3/L4 L4/L5 L5/S1
Lee (2014) [24]	South Korea	Retrospective case‐series	52	57.2 (11.3)	18/34	L5/S1
Papavero (2013) [25]	Germany	Prospective case‐series	22	64	15/7	^a^
Ryang (2007) [7]	Germany	Prospective case‐series	15	^a^	10/5	L3/L4 L4/L5 L5/S1
Salame (2010) [6]	Israel	Retrospective case‐series	31	52	15/16	L2/L3 L3/L4 L4/L5 L5/S1
Siu (2016) [26]	Australia	Prospective case‐series	19	58.4	10/9	L1/L2 L2/L3 L3/L4 L4/L5 L5/S1
Siu (2016) [27]	Australia	Prospective case‐series	24	57.2	11/13	L1/L2 L2/L3 L3/L4 L4/L5 L5/S1
Vogelsang (2008) [28]	Austria	Retrospective case‐series	59	58.3	39/34	L2/3 L3/4 L4/5 L5/S1

Abbreviation: SD, standard deviation.

^a^ Data not reported in the study.

### 3.3 Pain Assessed by VAS

Eleven studies utilized a VAS to assess preoperative pain and at least one timepoint of postoperative pain (Table 3). Eight studies measured lower back pain [5, 6, 17, 19, 22, 24, 26, 27], nine studies measured leg pain [5, 6, 17, 19, 20, 22, 23, 26, 27], and one study did not specify the type of pain measured [18].

**TABLE 3:** Assessment of pain outcomes using the visual analog scale in included studies.

Author (year)	Preoperative mean (SD) *T*_0_	Postoperative mean (SD) *T*_1_	Timing of *T*_1_ measurement	Follow‐up mean (SD) *T*_2_	Timing of *T*_2_ measurement	Follow‐up mean (SD) *T*_3_	Timing of *T*_3_ measurement	Follow‐up mean (SD) *T*_4_	Timing of *T*_4_ measurement	Follow‐up mean (SD) *T*_5_	Timing of *T*_5_ measurement
*Leg pain*											
Abdelgawaad (2018) [17]	7.6	1.4^a^	Immediately	1.2	12–54 months (M = 38)	—	—	—	—	—	—
Eicker (2013) [19]	7.9	1.3^a^, ^b^	6 months	—	—	—	—	—	—	—	—
Fuentes (2009) [20]	7	2	—	—	—	—	—	—	—	—	—
Greiner—Perth (2003) [5]	7	3.6^a^	Immediately	—	—	—	—	—	—	—	—
Kang (2024) [22]	7.64 (1.1)	2.85 (1.8)^a^, ^b^	2 days	2.30 (1.7)	1 month	1.78 (1.5)	3 months	1.52 (1.3)	6 months	1.52 (1.4)	1 year
Kogias (2007) [23]	8.5 (0.8)	1.5 (0.4)^a^	Immediately	—	—	—	—	—	—	—	—
Salame (2010) [6]	8.6	3.8^a^	Immediately	1.3	6 months	0.6	18–38 months (*M* = 25.16)	—	—	—	—
Siu (2016) [26]	5.5 (3.1)	1.7 (2.1)^a^, ^b^	6 weeks	2 (1.8)	3 months	1.8 (1.3)	6 months	—	—	—	—
Siu (2016) [27]	5.5 (2.9)	2.2 (2.4)^a^, ^b^	6 weeks	1.9 (1.8)	3 months	1.9 (1.5)	6 months	—	—	—	—
Lower back pain											
Abdelgawaad (2018) [17]	2.5	1.3^a^	Immediately	1	12–54 months (*M* = 38)	—	—	—	—	—	—
Eicker (2013) [19]	2.4	1.4^a^, ^b^	6 months	—	—	—	—	—	—	—	—
Greiner—Perth (2003) [5]	6.3	4.4^a^, ^b^	Immediately	—	—	—	—	—	—	—	—
Kang (2024) [22]	5.36 (1.2)	3.64 (0.8)^a^, ^b^	2 days	2.30 (1)	1 month	2.22 (0.9)	3 months	1.94 (1)	6 months	1.82 (1)	1 year
Lee (2014) [24]	7.6 (1.3)	3.6 (1.1)	Immediately	—	—	—	—	—	—	—	—
Salame (2010) [6]	5.8	4.8^a^	Immediately	0.8	6 months	0.7	18–38 months (*M* = 25.16)	—	—	—	—
Siu (2016) [26]	4.9 (2.8)	2.2 (2.2)^a^, ^b^	6 weeks	1.6 (1.8)	3 months	1.5 (1.5)	6 months	—	—	—	—
Siu. (2016) [27]	5 (2.8)	2.4 (2.4)^a^, ^b^	6 weeks	1.5 (1.8)	3 months	1.5 (1.5)	6 months	—	—	—	—
Unspecified pain											
Antony (2022) [18]	7.29 (1.2)	2.04 (1.5)^a^, ^b^	1 day	0.8 (1.6)	3 months	0.5 (1.6)	1 year	—	—	—	—

Abbreviations: *M*, mean; SD, standard deviation.

^a^ Statistically significant difference compared to prescores reported.

^b^ Analysis details provided.

The timing of the initial postoperative VAS assessments occurred either immediately or 1 day after surgery [5, 6, 17, 18, 23, 24], 2 days after surgery [22], at 6 weeks after surgery [26, 27], or 6 months after surgery [19], and was unspecified in one study [20]. Sample sizes of patients who provided pre‐ and postoperative data ranged from 15 to 76. Preoperative VAS scores for leg pain across the included studies ranged from 5.5 to 8.6, while lower back pain scores ranged from 2.4 to 7.6. Immediately or 1 day after surgery, postoperative VAS scores for leg pain ranged from 1.4 to 3.8, and for lower back pain, scores ranged from 1.3 to 4.8, though one study did not specify whether its reported postoperative VAS score referred to leg or lower back pain [5, 6, 17, 18, 23, 24]. Another study reported postoperative VAS scores at 2 days postsurgery of 2.85 for leg pain and 3.64 for lower back pain [22]. At 6 weeks after surgery, VAS scores for leg pain ranged from 1.7 to 2.2, and for lower back pain, from 2.2 to 2.4 [26, 27]. At 6 months, one study reported VAS scores of 1.3 for leg pain and 1.4 for lower back pain [19]. In another study, the timing of postoperative VAS measurement was not provided, though it reported a score of 2 for leg pain, with no corresponding score for lower back pain [20].

Nine studies reported significant differences between pre‐ and postscores for leg pain and lower back pain [5, 6, 17, 18, 19, 22, 23, 26, 27], but only six reported on the analytical tests that were conducted to demonstrate significance [5, 18, 19, 22, 26, 27]. In one of these studies, the authors noted that acute FLLDH patients had higher preoperative leg pain VAS scores than chronic FLLDH patients, potentially accounting for a greater observed improvement for the acute subgroup [19]. Two studies did not report significance, only improvement in scores [20, 24]. In one of these studies, the authors noted that although a decrease in mean lower back pain scores was observed after surgery, radicular symptoms persisted in two patients [24].

Six studies reported follow‐up scores on the VAS (Table 3), but none assessed the statistical significance of these follow‐up outcomes compared to pre‐ or postoperative scores. Postoperative VAS follow‐up scores were recorded at 1 month [22], 3 months [18, 22, 26, 27], 6 months [6, 22, 26, 27], and 1 year after surgery [18, 22]. Sample sizes of patients who provided follow‐up data ranged from 19 to 76. At 1 month postsurgery, one study reported scores of 2.3 for both leg and lower back pain [22]. Four studies provided data at 3 months, and in three of these studies, leg pain scores ranged from 1.78 to 2 and lower back pain scores from 1.5 to 2.22 [22, 26, 27], while one study reported an unspecified pain score of 0.8 [18]. Four studies provided data at 6 months, and in these studies, leg pain scores ranged from 1.3 to 1.9, and lower back pain scores ranged from 0.8 to 1.94 [6, 22, 26, 27]. Two studies reported follow‐up scores at 1 year; one study reported a leg pain score of 1.5 2 and a lower back pain score of 1.82 [22], while the other reported an unspecified VAS score of 0.5 [18]. One study reported follow‐up scores taken 12–54 (average 38) months after surgery, and scores were 1.2 for leg pain and 1 for lower back pain [17]. Another study, with a follow‐up at 18–36 months (average 25.2), reported scores of 0.6 for leg pain and 0.7 for back pain [6].

### 3.4 Mobility Assessed by Oswestry Disability Index

Seven studies assessed mobility outcomes postsurgery using the ODI (Table 4) [5, 17, 18, 19, 22, 26, 27]. All seven studies reported significant differences between pre and post scores, but one study did not specify the analytical test used to demonstrate significance [17].

**TABLE 4:** Oswestry Disability Index measures for mobility.

Author (year)	Preoperative mean (SD) *T*_0_	Postoperative mean (SD) *T*_1_	Timing of *T*_1_ measurement	Follow‐up mean (SD) *T*_2_	Timing of *T*_2_ measurement	Follow‐up mean (SD) *T*_3_	Timing of *T*_3_ measurement	Follow‐up mean (SD) *T*_4_	Timing of *T*_4_ measurement
Abdelgawaad (2018) [17]	42	12.3^a^	Immediately	10	12–54 months (*M* = 38)	—	—	—	—
Antony (2022) [18]	37.3 (14.8)	5.5 (8.4)^a^, ^b^	1 day	3.4 (8.4)	3 months	2.4 (7.6)	1 year	—	—
Eicker (2013) [19]	42	12.3^a^, ^b^	6 months	—	—	—	—	—	—
Greiner‐Perth (2003) [5]	30.6	14.3^a^, ^b^	Immediately	—	—	—	—	—	—
Kang (2024) [22]	56.7 (13.9)	30.3 (12.2)^a^, ^b^	1 month	24.6 (11.4)	3 months	21.7 (10.7)	6 months	20 (11.3)	1 year
Siu (2016) [26]	48.1 (20.9)	22.5 (13.9)^a^, ^b^	6 weeks	13.1 (11.8)	3 months	19.1 (13.3)	6 months	—	—
Siu (2016) [27]	46.2 (19.6)	24.6 (14.9)^a^, ^b^	6 weeks	16.3 (12.5)	3 months	18.2 (13.3)	6 months	—	—

Abbreviations: *M*, mean; SD, standard deviation.

^a^ Statistically significant difference compared to prescores reported.

^b^ Analysis details provided.

The timing of the initial postoperative ODI assessments occurred either immediately or 1 day after surgery [5, 17, 18], 1 month after surgery [22], 6 weeks after surgery [26, 27], or 6 months after surgery [19]. Sample sizes of patients who provided pre‐ and postoperative data ranged from 15 to 76. Preoperative ODI scores ranged from 30.6 to 56.7. Immediately or 1 day after surgery, ODI ranged from 5.5 to 14.3 [5, 17, 18]. At 1 month postsurgery, one study reported an ODI score of 30.3 [22]. At 6 weeks after surgery, ODI scores ranged from 22.5 to 24.6 [26, 27], while at 6 months postsurgery, one study reported an ODI score of 12.3 [19].

Five studies reported follow‐up ODI scores, but none assessed the statistical significance of these follow‐up outcomes compared to pre‐ or postoperative scores [17, 18, 22, 26, 27]. Postoperative follow‐up ODI scores were recorded at 3, 6, and 12 months after surgery. Four studies provided postoperative scores at 3 months, which ranged from 3.4 to 24.6 [18, 22, 26, 27], and three studies provided postoperative scores at 6 months, which ranged from 2.4 to 21.7 [22, 26, 27]. Two studies reported postoperative ODI scores at 1 year, which ranged from 2.4 to 20 [18, 22]. Additionally, one study reported follow‐up scores taken 12–54 (average 38) months postsurgery, with an ODI score of 10 [17].

### 3.5 Pain and Mobility Assessed by Modified MacNab Criteria

Seven studies utilized the Modified MacNab criteria to evaluate pain and mobility outcomes (Table 5) [7, 21, 22, 24, 25, 27, 28]. Assessments using the MacNab criteria occurred anywhere between 1 and 47 months after surgery. Only one study specified the timing of this assessment, as 6 months postsurgery [27]. The remaining studies reported the range of varying timepoints in which MacNab assessments were made. For example, in one study, assessments were conducted via telephone inquiries that occurred anywhere between 10 and 47 months after surgery [28].

**TABLE 5:** Modified MacNab criteria measures for pain and mobility.

Author (year)	Modified MacNab criteria (% of patients)	Sample size assessed	Timing of postoperative assessment
Hitchon (2015) [21]	Excellent (52%) Good (37%) Fair (0%) Poor (10%)	40	6 weeks–6 months
Kang (2024) [22]	Excellent (18.2%) Good (54.5%) Fair (18.2%) Poor (9.1%)	33	^a^
Lee (2014) [24]	Excellent (71%) Good (25%) Fair (3.8%) Poor (0%)	52	1–27 months (*M* = 7)
Papavero (2013) [25]	Excellent (45%) Good (23%) Fair (14%) Poor (18%)	22	22–41 months (*M* = 27)
Ryang (2007) [7]	Excellent (40%) Good (53%) Fair (7%) Poor (0%)	15	*M* = 24 months
Siu (2016) [27]	Excellent (67%) Good (29%) Fair (4%) Poor (0%)	24	6 months
Vogelsang (2008) [28]	Excellent (39%) Good (42.4%) Fair (13.5%) Poor (5.1%)	59	10–47 months

*Note*: “Excellent” indicates complete absence of pain and mobility restrictions, enabling patients to resume normal activities. “Good” indicates occasional pain with relief from preoperative symptoms, allowing for modified work. “Fair” denotes slight improvement in functional capacity, although patients remain handicapped. “Poor” indicates no postoperative improvement, necessitating further surgical intervention [29].

Abbreviation: *M*, mean.

^a^ Timing of postoperative assessment not specified.

Across all studies, 18.2%–71% of patients achieved excellent outcomes, 23%–54.5% achieved good outcomes, 0%–18.2% achieved fair outcomes, and 0%–18% achieved poor outcomes postsurgery [7, 21, 22, 24, 25, 27, 28]. In one study, two patients reported severe pain with a severity rating of 9/10 at 1 and 6‐month follow‐ups [21].

### 3.6 Secondary Outcomes

Secondary outcomes are described below and provided in tabular form in Appendix B in the Supporting Information.

#### 3.6.1 Blood Loss

Four studies reported data on blood loss resulting from MTD procedures [7, 21, 23, 24]. Among them, three studies reported mean operative blood loss, ranging from 30 to 70 ml [21, 23, 24]. Of these studies, one reported a blood loss range of 25–300 mL [24], whereas the two other studies reported a smaller variation in blood loss between participants, at 30 ± 20 and 37 ± 31 mL, respectively [21, 23]. In one study, blood loss was reported to be less than 20 mL among all patients who underwent MTD, but no average volume was reported [7]. In two studies, the authors mentioned that blood loss was minimal, but no numerical data was provided [6, 17].

#### 3.6.2 Operation Time

Twelve studies reported the operation time for the MTD procedure [5, 6, 7, 17, 19, 20, 21, 22, 23, 24, 27, 28]. Mean operative time was reported in 11 studies and ranged from 43 to 126 min [5, 6, 7, 17, 19, 20, 21, 22, 23, 24, 28]. Seven studies reported the range of operation time in minutes [6, 7, 17, 19, 20, 24, 27]. Two notably large ranges were 25–114 min [17] and 25–180 min [19].

#### 3.6.3 Hospital Stay Duration and Return to Daily Activities

Eleven studies reported on hospital duration after MTD [5, 6, 7, 18, 20, 21, 22, 23, 25, 26, 27]. Five studies reported the average hospital stay duration, which ranged from one to four days [6, 18, 20, 21, 25]. Eight studies reported the range (in days) of hospital stay, which was between 1 and 10 days [5, 6, 7, 20, 22, 23, 26, 27]. Three studies reported the latency period for patients to resume daily activities [5, 23, 28]. One study reported that most patients resumed work within 4–8 weeks postoperation [28], while another study reported the same latency to work period of 4–8 weeks but specifically for employed patients only [5], Kogias et al. reported all patients were discharged by the third day and resumed daily activities, but the timeframe was not specified [23].

#### 3.6.4 Reherniation and Reoperation

Six studies reported instances of patients experiencing reherniation [5, 7, 17, 19, 22, 28]. In two studies, only one patient had reherniation [5, 7], in three studies, two patients had reherniation [17, 19, 28], and in one study, four patients had reherniation [22]. Seven studies reported on patients that required reoperations using MTD [5, 6, 17, 18, 19, 23, 28]. These reoperations were undertaken either to resolve recurrent herniation, infection, or residual herniated tissue from the original procedure.

Peri‐Operative and Postoperative Complications

Six studies documented peri‐ and postoperative complications [6, 17, 21, 22, 24, 28]. Four studies reported either one or two patients having complications [6, 17, 21, 28], while one study reported seven [22], and another study reported that 15 out of 70 patients had complications, most of which resolved within a month [24]. Eight studies reported no complications following surgery. One study did not provide information regarding complications [25].

### 3.7 Quality Assessment

All 15 studies were appraised using the JBI Critical Appraisal Checklist for either Case Series or Cohort Studies. Details of quality appraisal can be found in Appendix C in the Supporting Information.

Overall, across all the case series, there was missing information in at least two of the examined areas, with one study failing to satisfy any of the criteria [25]. The lowest scoring areas were standardization and reliability of condition measurement (JBI Question #2), the consecutive inclusion and enrolment of participants (JBI Question #4 and #5), and the reporting of presenting site(s)/clinic(s) demographic information (JBI Question #9), with 10 studies failing to meet each of these criteria. About half of the case series studies did not clearly report the inclusion criteria (JBI Question #1), and four studies incompletely reported outcomes and follow‐up results, including standard deviations of mean scores and time points of measurement (JBI Question #8). High scoring criteria were the reporting of participants' clinical information, demonstrated in 11 studies (JBI Question #7), and the reporting of participant demographics, demonstrated in eight studies (JBI Question #6). The highest score on the JBI Case Series checklist was seven out of the 10 criteria, achieved by three studies [17, 19, 27].

The cohort study was of moderate quality, meeting key criteria for population comparability (JBI Question #1), outcome measurement (JBI Question #3), and statistical analysis (JBI Question #11), but it lacked clarity on surgical technique selection (JBI Question #2), confounding factors (JBI Question #5), and follow‐up completeness (JBI Question #9).

## 4 Discussion

Treatments for FLLDH are more challenging than treatments for common posterolateral disc herniations, as conventional open surgery provides limited access to the far lateral disc space [22]. MTD utilizes a minimally invasive paraspinal surgical corridor to provide direct access to the foraminal and extraforaminal zones affected by the disc herniation, making it a superior alternative to the conventional midline approach [22]. MTD has demonstrated potentially safer lateral nerve root exposure and extraforaminal decompression while preserving the facet joints and reducing muscle trauma, contributing to faster recovery and shorter hospital stays in comparison to conventional techniques [1, 30]. In this systematic review, we critically evaluated 15 studies on the use of MTD for FLLDH, and the collective evidence supports the safety and effectiveness of MTD for treating patients with FLLDH.

### 4.1 Pain and Mobility

Nine studies reported that MTD significantly reduced leg and lower back pain in FLLDH patients postsurgery [5, 6, 17, 18, 19, 22, 23, 26, 27]. According to Ostelo et al., a clinically meaningful improvement in lower back pain postsurgery is indicated by a minimum of 20 mm reduction on the VAS [31]. This threshold is widely cited in studies on various discectomy techniques, lending support to its credibility in clinical settings [26, 32, 33]. Of the eight studies reporting improved lower back pain postsurgery, four studies demonstrated clinically meaningful improvements in pain over the follow‐up duration using this criterion [22, 24, 26, 27]. Similarly, according to Ostelo et al., a clinically meaningful improvement in mobility postsurgery is indicated by at least a 10‐point improvement on the ODI [31]. Seven studies reported that MTD significantly improved mobility in FLLDH patients postsurgery, and all seven of these studies demonstrated clinically meaningful improvement according to Ostelo's criterion [5, 17, 18, 19, 22, 26, 27].

When comparing pain and mobility outcomes between MTD and other techniques like endoscopic discectomy (ED), similar postsurgical improvements measured by the VAS, ODI, and MacNab criteria are evidenced [34, 35, 36]. The cohort study by Kang et al., the only included study with a matched comparison group, reported that biportal ED led to significantly reduced VAS scores for lower back pain than MTD at 2 days postsurgery [22]. However, this advantage was not sustained in later follow‐ups, indicating that ED may provide early pain relief but no long‐term benefit over MTD. Additionally, Kang et al. reported similar percentages of excellent or good MacNab outcomes for ED and MTD, consistent with the MTD studies in this review, suggesting that both techniques are similarly effective in improving pain and mobility in FLLDH patients [22].

### 4.2 Secondary Outcomes

Secondary outcomes provided further insight into the potential effectiveness of MTD. Operative times, blood loss, hospital stay duration, and early mobilization outcomes reported in the included studies were comparable to those reported for other minimally invasive surgical techniques. For example, although operative times varied considerably across the included studies, these operative time ranges are similar to those reported in studies examining ED [34, 35, 37]. The cohort study by Kang et al. demonstrated no statistically significant difference in operative times between MTD and ED [22]. Blood loss, hospital stay duration, and early mobilization outcomes of MTD, as evidenced in the included studies, are also comparable to those reported for endoscopic techniques [35, 36]. This provides further evidence that MTD may be as effective in promoting rapid recovery and a quick return to daily activities when compared to similar techniques [34, 35, 37].

In terms of surgical complications, most research on ED for FLLDH shows comparable surgical outcomes to those of the MTD studies included in this review, with minimal complications [34, 35, 36]. However, in Kang et al.'s study, the MTD group had a significantly higher reoperation rate and slightly more complications than the ED group [22]. The authors suggested this was possibly due to increased posterior muscle damage and facet violations in MTD, which may lead to segmental instability and a higher risk of local recurrence compared to ED [22]. Due to the limited number of studies comparing complications of MTD when compared to other surgical techniques, it is difficult to generalize this finding.

ED is a valuable alternative to MTD but has a steeper learning curve, higher cost, and risks such as inadequate disc exposure, incomplete decompression, and nerve injury [37, 38]. In contrast, MTD offers better surgical visualization, particularly stereo vision, a larger operative channel that can accommodate conventional surgical instruments, and a safer transition from the standard open approach [22, 39]. When considering the potential benefits of ED, it is therefore important to question their clinical meaningfulness and carefully weigh them against the technical advantages of MTD that support the principles of safe surgery.

### 4.3 Limitations of Included Studies and Future Research Directions

It is important to acknowledge the limitations of the studies included in this review. The overall quality of the included studies was low to moderate, undermining the validity and reliability of the reported improvements. First, although many studies included in this review met Ostelo's criteria for clinical improvement, most studies were case series designs, which are prone to selection bias [40]. There is minimal detail reported in these studies about inclusion and exclusion criteria for participants, providing little information about whether study participants were representative of patients with FLLDH who undergo MTD. Furthermore, the paucity of directly comparative studies between MTD and ED, and the absence of comparison groups in most series, limits accurate estimation of MTD's effectiveness and renders a formal meta‐analysis of MTD versus ED infeasible. To address these issues, future research should prioritize employing matched comparison studies with adequately powered sample sizes and systematic patient selection methods to generate the consistent, comparable data necessary for meaningful meta‐analyses.

Second, most studies examined short‐term postoperative outcomes, with only two studies providing 12‐month follow‐up data [18, 22]. These studies showed continued improvements in VAS and ODI scores over time, indicating the potential sustainability of improved outcomes resulting from MTD [18, 22]. It is critical that future research employs longitudinal designs with extended follow‐up periods to test the replicability of these studies and thus the durability of MTD's benefits.

Third, scores on the VAS and ODI reflect patients' self‐reported experience of pain and physical functioning, which is inherently subject to reporting bias associated with subjective measures [41]. These may include factors such as patients' mood, expectations, interactions with the healthcare providers, and socioeconomic situation, leading to fluctuations that are difficult to control [42]. By matching patient groups based on similar baseline characteristics and clinical conditions, differences in outcomes can be more confidently attributed to the effects of MTD rather than to confounding factors [42, 43, 44]. Furthermore, increasing the frequency of measurement at multiple time points would provide a more comprehensive understanding of symptom progression and recovery trajectories, minimizing the impact of single‐time‐point variations that may result from temporary fluctuations in a patient's condition [42].

## 5 Conclusion

This review contributes to the growing evidence base on the use of MTD for FLLDH, providing further insight into its safety and effectiveness as a treatment option. However, methodological limitations of the reviewed studies, including small sample sizes, variable follow‐up periods, and lack of adequate controls, constrain definitive conclusions about MTD's superiority over other discectomy techniques.

## Author Contributions

**Chamath Jagoda:** conceptualization, methodology, validation, investigation, formal analysis, resources, writing – original draft, writing – review and editing, visualization. **Samantha Spanos:** conceptualization, methodology, validation, investigation, formal analysis, resources, writing – original draft, writing – review and editing, supervision. **Timothy L. Siu:** conceptualization, resources, supervision. All authors had full access to the study data and accept responsibility for the integrity and accuracy of the data and the accuracy of the analysis. All authors meet the authorship criteria and are in agreement with the manuscript.

## Conflicts of Interest

The authors declare no conflicts of interest.

## Supporting information


**Appendix A.** Search strategy.

**Appendix B.** Secondary outcomes measures in the included studies.

**Appendix C.** Quality assessment of included studies according to the JBI critical appraisal checklist.

## Data Availability

The data that support the findings of this study are available from the corresponding author upon reasonable request.
