# Supplementary material for: Effectiveness of Microscopic Tubular Discectomy for Improved Pain and Mobility in Far Lateral Lumbar Disc Herniation: A Systematic Review
Source: Orthop Surg. 2025 Oct 12;17(12):3289–301. doi: 10.1111/os.70187 (PMC12685468; doi:10.1111/os.70187)
Supplement: Supplementary file 1 — Appendix A. Search strategy. [file OS-17-3289-s003.docx]

**Appendix A:** Search strategy

(Lumbar AND (Discectomy OR Diskectomy OR Microdiscectomy OR Microdiskectomy) AND (tubul* OR Wiltse OR Paramedian OR Para-median OR Para-spinal OR Paraspinal OR Transmuscular OR Trans-muscular OR Para-muscular OR Trans-tubular OR Transtubular OR METRx OR Muscle-sparing OR Muscle-splitting OR Dilator OR Channel) AND Microscop*)
