# Supplementary material for: Effectiveness of Microscopic Tubular Discectomy for Improved Pain and Mobility in Far Lateral Lumbar Disc Herniation: A Systematic Review
Source: Orthop Surg. 2025 Oct 12;17(12):3289–301. doi: 10.1111/os.70187 (PMC12685468; doi:10.1111/os.70187)
Supplement: Supplementary file 2 — Appendix B. Secondary outcomes measures in the included studies. [file OS-17-3289-s002.docx]

**Appendix B:** Secondary outcomes measures in the included studies.

| **Author (year)** | **Operation time in minutes**  ***M (SD)^2^, range*** | **Duration of hospital stay** | **Blood loss in ml**  ***M (SD)^2^*** | **Return to daily activities** | **Re-herniation / Re-operation** | **Peri-operative and post-operative complications** |
| --- | --- | --- | --- | --- | --- | --- |
| *Abdelgawaad*  *(2018)^17^* | 57.5,  25 - 114 | - | - | - | Two patients (2.6%) had a re- herniation. They had a successful reoperation. | One patient (1%) had postoperative quadricep weakness which resolved after 3 months. Another patient had deep vein thrombosis and were admitted to hospital. |
| *Antony (2022)^18^* | - | 1 day | - | - | Two patients (7%) had recurrent and persistent radicular pain. One of them had a successful redo MTD^3^. | None. |
| *Eicker (2013)^19^* | 67.5,  25 - 180 | - | - | - | Two patients (4%) had recurrences of disc herniation and had a reoperation with MTD^3^. | None. |
| *Fuentes (2009)^20^* | 55,  45 - 90 | Average of 3 days (range: 1 – 5) | - | - | - | None. |
| *Greiner-Perth (2003**)^5^* | 43 | 3 to 4 days | - | All employed patients returned to work within 4-8 weeks post-surgery. | One patient (6.7%) had recurrent disc herniation after 2 months which was treated with MTD^3^. | None. |
| *Hitchon (2015)^21^* | 126 (34) | Average of 1 ± 1 day | 37 (31) | - | - | Two patients (5%) developed chronic back pain. One patient was re-admitted for a candida wound infection. |
| *Kang (2024)^22^* | 90.76 (35.64) | 2 to 3 days | - | - | Four patients (12.1%) had recurrences of local FLLDH and underwent transforaminal lumbar interbody fusion. | Two patients (6.1%) developed surgical site infections, one (3%) had an epidural hematoma, three (9.1%) experienced postoperative dysesthesia, and one (3%) had delirium. |
| *Kogias (2007)^23^* | 51 (15) | 1 to 3 days | 30 (20) | All patients resumed daily activities. | One patient (3%) had poor pain improvement due to insufficient removal of herniated tissue. This was resolved through a revision surgery with MTD^3^. | None. |
| *Lee (2014)^24^* | 75,  45 – 200 | - | 70 | - | - | Nine patients (17%) had mild postoperative dysesthesias, and six (12%) had moderate postoperative dysesthesias. |
| *Papavero*  *(2013)^25^* | - | 4 days | - | - | Four patients (18%) underwent second surgery for pedicle screw fixation and fusion. | - |
| *Ryang (2007)^7^* | 75,  29 - 109 | Median of 4 days (range: 3-10) | - | - | One patient (7%) had recurrent LDH^4^ at the operated level. Another patient (7%) had residual herniated tissue. No re-operation occurred for both patients. | None. |
| *Salame (2010)^6^* | 44,  30 – 65 | Average of 1.3 days (range: 1 – 5) | - | - | One patient (3%) had post-operative residual disc herniation which was resolved through a repeat MTD^3^. | Two patients (6%) had a dural tear which was re-sutured with no postoperative CSF^5^ leakage. |
| *Siu (2016****a****)^26^* | - | 1 to 2 days | - | - | - | None. |
| *Siu (2016****b****)^27^* | 60 – 120 | 1 to 2 days | - | - | One patient (4%) had persistent self-limiting symptoms 12 months postoperatively; no re-operation occurred. | None. |
| *Vogelsang (2008)^28^* | 58 | - | - | Of the 24 employed patients, 20 resumed work within 4 – 8 weeks post-surgery. | Two patients (4%) had re-herniations requiring re-operation with MTD^3^, and one patient had a re-operation due to a deep wound infection. Another two patients had unspecified spinal surgery due to herniations in other disc levels. | Two patients (4%) experienced self-limiting allodynia. One patient suffered a deep wound infection. |

*^1^M, mean; ^2^SD, standard deviation; ^3^MTD, microscopic tubular discectomy; ^4^LDH, lumbar disc herniation; ^5^CSF, cerebrospinal fluid.*
