# Supplementary material for: Effectiveness of Microscopic Tubular Discectomy for Improved Pain and Mobility in Far Lateral Lumbar Disc Herniation: A Systematic Review
Source: Orthop Surg. 2025 Oct 12;17(12):3289–301. doi: 10.1111/os.70187 (PMC12685468; doi:10.1111/os.70187)
Supplement: Supplementary file 3 — Appendix C. Quality assessment of included studies according to the JBI critical appraisal checklist. [file OS-17-3289-s001.docx]

**Appendix C:** Quality assessment of included studies according to the JBI critical appraisal checklist.

| **Author (year)** | **Q1** | **Q2** | **Q3** | **Q4** | **Q5** | **Q6** | **Q7** | **Q8** | **Q9** | **Q10** | **Q11** |
| --- | --- | --- | --- | --- | --- | --- | --- | --- | --- | --- | --- |
| ***Checklist for case series*** | | | | | | | | | | | |
| *Abdelgawaad*  *(2018)^17^* | Yes | No | Yes | Yes | Yes | Yes | Yes | No | Yes | Unclear | N/A |
| *Antony (2022)^18^* | Yes | Unclear | Unclear | Yes | Unclear | Yes | Yes | Yes | Yes | Unclear | N/A |
| *Eicker (2013)^19^* | Yes | Yes | Unclear | No | Yes | Yes | Yes | Yes | No | Yes | N/A |
| *Fuentes (2009)^20^* | No | No | Unclear | No | Unclear | Yes | Yes | No | No | No | N/A |
| *Greiner-Perth (2003)^5^* | No | No | Unclear | No | Unclear | Yes | Yes | Yes | No | Yes | N/A |
| *Hitchon (2015)^21^* | No | No | Yes | No | Unclear | Yes | Yes | No | No | No | N/A |
| *Kogias (2007)^23^* | No | Yes | Yes | Unclear | Unclear | No | No | Yes | No | No | N/A |
| *Lee (2014)^24^* | No | No | Yes | Yes | Unclear | Yes | Yes | Yes | Yes | No | N/A |
| *Papavero*  *(2013)^25^* | No | No | Unclear | No | No | No | No | No | No | No | N/A |
| *Ryang (2007)^7^* | No | No | Yes | Unclear | Unclear | Yes | Yes | Yes | No | No | N/A |
| *Salame (2010)^6^* | No | Yes | Yes | Yes | Unclear | Yes | Yes | Yes | No | No | N/A |
| *Siu (2016****a****)^26^* | Yes | Unclear | Yes | No | Yes | No | Yes | Yes | No | Yes | N/A |
| *Siu (2016****b****)^27^* | Yes | Yes | Unclear | No | Yes | Yes | Yes | Yes | No | Yes | N/A |
| *Vogelsang. (2008)^28^* | No | No | Yes | Unclear | Unclear | Yes | Yes | No | No | No | N/A |
| ***Checklist for cohort studies*** | | | | | | | | | | | |
| *Kang (2024)^22^* | Yes | No | Yes | No | No | Unclear | Yes | Yes | No | Unclear | Yes |

*JBI, Joanna Briggs Institute.* ***Checklist for case series*** *Q1: Were there clear criteria for inclusion in the case series? Q2: Was the condition measured in a standard, reliable way for all participants included in the case series? Q3Were valid methods used for identification of the condition for all participants included in the case series? Q4Did the case series have consecutive inclusion of participants? Q5Did the case series have complete inclusion of participants? Q6Was there clear reporting of the demographics of the participants in the study? Q7Was there clear reporting of clinical information of the participants? Q8Were the outcomes or follow up results of cases clearly reported? Q9Was there clear reporting of the presenting site(s)/clinic(s) demographic information? Q10Was statistical analysis appropriate?* ***Checklist for cohort studies*** *Q1: Were the two groups similar and recruited from the same population? Q2: Were the exposures measured similarly to assign people to both exposed and unexposed groups? Q3: Was the exposure measured in a valid and reliable way? Q4: Were confounding factors identified? Q5: Were strategies to deal with confounding factors stated? Q6: Were the groups/participants free of the outcome at the start of the study (or at the moment of exposure)? Q7: Were the outcomes measured in a valid and reliable way? Q8: Was the follow up time reported and sufficient to be long enough for outcomes to occur? Q9: Was follow up complete, and if not, were the reasons to loss to follow up described and explored? Q10: Were strategies to address incomplete follow up utilized? Q11: Was appropriate statistical analysis used?*
